# Questing for the identity of *Hepatozoon* in foxes

**DOI:** 10.1186/1756-3305-7-S1-O23

**Published:** 2014-04-01

**Authors:** B Mitková, MA Qablan, AD Mihalca, D Modrý

**Affiliations:** 1 Department of Pathological Morphology and Parasitology, University of Veterinary and Pharmaceutical Sciences Brno, Czech Republic; 2 Department of Parasitology and Parasitic Diseases, Faculty of Veterinary Medicine, University of Agricultural Sciences and Veterinary Medicine Cluj-Napoca, Romania; 3 Institute of Parasitology, Biology Centre, and Faculty of Sciences, University of South Bohemia, České Budějovice (Budweis), Czech Republic

## 

Apicomplexan parasites of genus *Hepatozoon *invade the blood cells of many mammalian species, being transmitted by range of arthropods. In domestic carnivores, three species of *Hepatozoon *were described to date: *H. canis *and *H. americanum *in dogs and *H. felis *in cats. The classification of *Hepatozoon *in wild carnivores is still not complete due to lack of field and experimental data as well as phylogenetic studies. The aim of this study is to carry out a survey on the prevalence and diversity of *Hepatozoon *sp. in red foxes *Vulpes vulpes*. Samples of tissues were collected from dead foxes in 11 counties of Romania; 91 samples of liver tissue were examined in total. DNA extraction was performed with commercial kit according to the manufacture's protocol. *Hepatozoon *sp. DNA was detected by PCR using primers amplifying 400-600 bp long part of 18S rRNA gene. These primers are commonly used for diagnostic purposes in dogs and in some studies also for detection of *Hepatozoon *sp. in wild carnivores. PCR products were sequenced to validate positive results of reaction. DNA of parasite was confirmed in 55% of examined samples. Recent findings classified *Hepatozoon *sp. in foxes and other wild canids in Europe as *H. canis*. However, *Rhipicephalus sanguineus*, the only known vector of *H. canis *in Europe, is absent in most of our sampling sites. Moreover, this tick is typical for the dogs but rare or even absent in foxes. In order to clarify the identity of the parasite, the next step of our study is to focus on amplification of longer or full segment of 18s rRNA gene to allow more accurate phylogenetic analyses and comparison with *H. canis *sequences from dogs and *Hepatozoon *isolates from other carnivores.

This study was supported by IGA UVPS Brno, project 115/2013/FVL.

